# Hypodontia: An Update on Its Etiology, Classification, and Clinical Management

**DOI:** 10.1155/2017/9378325

**Published:** 2017-03-19

**Authors:** Azza Husam Al-Ani, Joseph Safwat Antoun, William Murray Thomson, Tony Raymond Merriman, Mauro Farella

**Affiliations:** ^1^Department of Oral Sciences, Faculty of Dentistry, University of Otago, Dunedin, New Zealand; ^2^Department of Biochemistry, Faculty of Dentistry, University of Otago, Dunedin, New Zealand

## Abstract

Hypodontia, or tooth agenesis, is the most prevalent craniofacial malformation in humans. It may occur as part of a recognised genetic syndrome or as a nonsyndromic isolated trait. Excluding third molars, the reported prevalence of hypodontia ranges from 1.6 to 6.9%, depending on the population studied. Most affected individuals lack only one or two teeth, with permanent second premolars and upper lateral incisors the most likely to be missing. Both environmental and genetic factors are involved in the aetiology of hypodontia, with the latter playing a more significant role. Hypodontia individuals often present a significant clinical challenge for orthodontists because, in a number of cases, the treatment time is prolonged and the treatment outcome may be compromised. Hence, the identification of genetic and environmental factors may be particularly useful in the early prediction of this condition and the development of prevention strategies and novel treatments in the future.

## 1. Definitions and Classifications

Hypodontia is the most prevalent dentofacial malformation in humans [1]. It may occur as part of a recognised genetic syndrome or as a nonsyndromic isolated trait [2]. The condition refers to the developmental failure of six or fewer teeth [3]. Its phenotypic presentation is varied in terms of severity and, as a result, various terms have been used to describe it. These terms include “congenitally missing teeth,” “tooth agenesis,” “hypodontia,” “oligodontia,” and “anodontia.” The term “congenitally missing teeth” is challenging because tooth development is completed after birth, so that the presence of most tooth germs can be proved only during childhood [4–6]. Tooth agenesis, on the other hand, refers directly to the developmental failure of a tooth. Other terms, such as hypodontia, are more suitable for classifying the type of tooth agenesis present and may be more appropriate in this context [7]. Oligodontia and anodontia are used to describe more severe forms of tooth agenesis, typically the absence of more than six teeth and the entire dentition [3], respectively. Tooth agenesis and hypodontia are the preferred terms in this work, with the latter term limited to missing teeth other than third molars.

## 2. Prevalence

### 2.1. Deciduous Dentition

Tooth agenesis is considered rare in the deciduous dentition and is not as common as in the permanent dentition. An association exists between hypodontia in the primary and permanent dentitions, with reports of children with primary teeth hypodontia showing absence of the corresponding successor teeth [8, 9]. A prevalence of less than 1% has been described in Caucasian populations [4], although it has been reported to be much higher in Japanese populations [10]. The prevalence of tooth agenesis in New Zealand appears to be consistent with that seen in Europe [11]. The deciduous maxillary lateral and mandibular central incisors account for 50% to 90% of affected deciduous teeth [4]. Most cases present as unilateral hypodontia, with mostly one or two teeth missing [8]. No significant sex difference in prevalence has been reported from any of the populations studied [8].

### 2.2. Permanent Dentition

The prevalence of hypodontia, which may be increasing with time, ranges from 1.6% to 36.5%, depending on the population studied [1]. At least 1 in 5 individuals lacks a third molar, while most individuals with hypodontia (80%) lack only one or two teeth [13, 14]. A meta-analysis investigated the prevalence of nonsyndromic tooth agenesis, included 33 studies from North America, Australia, and Europe, and found a higher prevalence in Europe (5.5%) and Australia (6.3%) than in North America [15]. Most individuals were missing only one or two permanent teeth, with very few missing more than six. Mandibular second premolars and the maxillary lateral incisors were reported to be the most likely to be missing [15, 16]. Notably, the prevalence of tooth agenesis in the last few decades has reportedly increased [17]. However, there is no empirical evidence to support whether this apparent increase is due to more advanced screening and diagnosis or other factors.

Hypodontia is typically associated with a number of classical features, including the site of agenesis and the size of the adjacent teeth. Tooth agenesis does not seem to affect the maxilla and the mandible differently [15], although there was one early study that found the mandible to be more frequently affected than the maxilla [18]. Comparing bilateral and unilateral agenesis, Polder et al. (2004) found that bilateral agenesis of maxillary lateral incisors occurred more often than unilateral agenesis. For the other teeth, such as the second mandibular premolar, unilateral agenesis was more common [15]. There appears to be no significant sex difference in missing primary teeth [19], although, in the permanent dentition, there seems to be a small albeit nonsignificant predilection of hypodontia in females [20]. One meta-analysis, however, found a significant difference in females, with the prevalence of hypodontia being 1.4 times higher in them than in males [15].

## 3. Features Associated with Hypodontia

Tooth agenesis is often nonsyndromic, but it can also be associated with oral clefts and several other syndromes [8]. For example, hypodontia is a common trait in cleft-lip and/or palate (CLP) patients [21]. The prevalence of hypodontia is higher in more severe clefting cases, most likely presenting with the agenesis of a maxillary lateral incisor (in either dentition) [4, 8]. In these patients, hypodontia in regions outside the cleft field is also more common than in the general population [22]. Other conditions that have hypodontia as one of their features include Down's Syndrome and ectodermal dysplasia. In these syndromes, there is a characteristic pattern of agenesis that is usually different from the overall population [4]. Moreover, recent data suggests that hypodontia shares some common pathways with particular kinds of cancer [23]

It is not known whether individuals with hypodontia have characteristic skeletal features and growth patterns, although some evidence suggests that hypodontia patients have significantly different craniofacial features from those with no missing teeth [24]. What is known is that tooth agenesis, especially in its severe forms, contributes to abnormal occlusion and is often associated with various anomalies in other teeth [4]. These include delays in development, ectopic eruption, reduction in tooth dimensions and morphology, shortened roots, taurodontia, and enamel hypoplasia [8].

### 3.1. Dental Features

Microdontia is a widely reported feature of hypodontia in case reports and case series [19]. This condition, which can affect one or more teeth, may be seen in either dentition [24, 25]. In addition, microdontia is genetic and presents in its severest form as ectodermal dysplasia [24]. It is also present in patients who have had chemotherapy or radiation of the jaws earlier in childhood [26]. Brook proposed that microdontia and hypodontia are linked genetically as a continuum of tooth size, where a tooth will fail to develop if the tooth germ does not reach a particular tooth size and tooth number “thresholds” [27].

Delays in tooth development are another common feature, whereby the absence of a permanent successor delays the normal resorption of the roots of the primary teeth. Indeed, the deciduous teeth may be retained for up to 40 or 50 years [28]. Meanwhile, approximately 46% of individuals with tooth agenesis also have short roots of other permanent teeth [8]. In addition, an association between taurodontism and hypodontia was found in a Dutch study, where taurodontism of the lower first molars was present in 29% of oligodontia patients but only 10% of controls [29].

**Figure 1:**
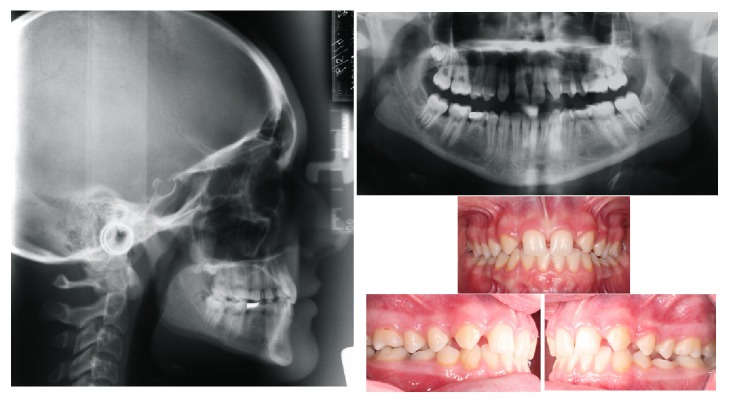
A female patient presenting with several common features of hypodontia. Note the agenesis of the maxillary lateral incisors and the second premolars, the retained primary mandibular molars, the generalised spacing, and the deep bite.

Another common feature of hypodontia is the ectopic positioning of the permanent teeth. This is likely caused by the absence of neighbouring teeth available to guide them during eruption or by the lack of space for them to erupt into. Transposition of teeth is also seen more commonly in individuals with hypodontia [30]. Tooth agenesis is also associated with enamel hypoplasia, diminutive or peg maxillary lateral incisors, primary molar infraocclusion, and palatally inclined or impacted maxillary canines [31, 32]. Intraorally, retroclined and overerupted lower incisors contribute to a greater overbite [33]. Generalised spacing and rotations of teeth adjacent to missing mandibular second premolars are also commonly seen [31]. Some of these features are evident in Figure 1.

### 3.2. Skeletal Features

Hypodontia patients tend to present with lower mandibular plane angles, associated with a smaller lower anterior face height and lip protrusion [34]. Other features include smaller maxillary and mandibular lengths and a Class III skeletal relationship tendency [35]. The short face height, along with the large freeway space, which is typical of hypodontia patients, may make them appear overclosed [24]. It was initially reported that children with hypodontia present with a shorter and more retrusive upper arch with proclined upper incisors [18]. However, the children were reexamined in another study and the authors reported that there were no changes in the craniofacial structures from 9 to 16 years of age to children without hypodontia [36].

In general, dentofacial changes are prominent in individuals with oligodontia, and these are related more to dental and functional compensation and not to a specific underlying pattern of growth [24, 35].

## 4. Aetiology

Numerous concepts about the aetiology of hypodontia have been proposed in the literature. The multiplicity of tooth agenesis theories suggests a multifactorial aetiology that involves genetic regulation and environmental factors. As such, the multifactorial nature of tooth agenesis entails a brief overview of tooth development and its genetic regulation. This will be followed by an outline of the theories surrounding hypodontia and a more detailed discussion of the specific factors, both genetic and environmental, that have been connected with this condition.

### 4.1. Tooth Development

Dental development is a complex process which involves mutual interactions between the oral epithelium and ectomesenchyme derived from the neural crest. During the initiation stage, thickening of the epithelium occurs, as it invaginates into the mesenchyme, creating a tooth bud [37]. Within the tooth bud, there is a collection of cells, the primary enamel knot, and these cells manage this process via signalling proteins. The mesenchyme surrounds the epithelium producing a cap stage, followed by a bell stage. Neighbouring mesenchymal cells differentiate into odontoblasts, and these secrete an organic dentine matrix [24]. Into this matrix, hydroxyapatite crystals are deposited [24]. At this stage, epithelial cells near to the dentine differentiate into ameloblasts, and these secrete an enamel matrix while controlling enamel mineralisation and maturation [37]. Secondary enamel knots control cusp formation in premolars and molars [38].

The region of the crown then undergoes histodifferentiation which is continued in the root. In terms of root development, apical extension of the odontogenic epithelium forms Hertwig's root sheath, which controls radicular dentine formation. This subsequently degenerates leading to cementoblast development. Following this, the cementoblasts produce cementum on the root [39]. Meanwhile, osteoblasts and fibroblasts, which aid in periodontal ligament formation, are produced from the differentiation of cells present in the dental follicle [40].

A series of genetically controlled successive molecular interactions are involved in the development of teeth [41, 42]. Numerous factors, such as those from the fibroblast growth factor (Fgf), wingless related integration site (Wnt), bone morphogenic protein (Bmp), and hedgehog (Hh) families, take part in the signalling of epithelial-mesenchymal interactions in tooth development [40]. Alterations in one or more of the signalling pathways may affect dental development and may play a role in causing a condition such as hypodontia.

### 4.2. Tooth Agenesis Theories

Several theories exist to decipher the cause of hypodontia, and most have focused on either genetic or environmental factors, although the importance of both components in the agenesis of teeth is now well recognised. These theories can be considered as either* evolutional* or* anatomical* [42].

Earlier studies concentrated on the evolutional viewpoint, which attributed tooth agenesis to shortening of the intermaxillary complex and the reduction in tooth number due to shorter arches. For instance, in 1945, Dahlberg used Butler's Field Theory that focused on evolution and development of mammalian teeth into the human dentition in order to explain different patterns of agenesis. Four morphological fields (incisors, canines, premolars, and molars) were described in each jaw. The more mesial tooth in each field was proposed to be the more genetically stable and as a result was seldom absent [24], while the teeth at the end of each field were less genetically stable. A later theory hypothesised that the last of each “class” were “vestigial bodies” that became obsolete during the evolution process [43]. Most currently, there is a theory that evolutionary change is working to reduce the human dentition by the loss of an incisor, premolar, and molar in each quadrant. According to Vastardis (2000), as humans evolve, the size of the jaws and the numbers of teeth appear to be decreasing [13].

Other theories focused on an anatomical principle, based on the hypothesis that specific areas of the dental lamina are prone to environmental effects throughout tooth maturation [42]. In support of this hypothesis, Svinhufvud et al. (1988) related the agenesis of the maxillary lateral incisors, the mandibular second premolars, and central incisors to the fact that they develop in areas of initial fusion of the jaw [44]. For example, maxillary lateral incisors develop in the region where the lateral maxillae and medial nasal bone processes fuse, while the mandibular second premolars originate in another delicate region [44]. Instead, Kjaer et al. (1994) argued that the region where development of innervation is last is the most sensitive one [45].

**Figure 2:**
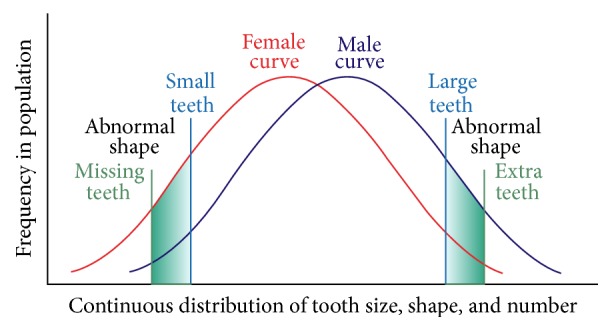
Model showing continuous distribution of tooth size, shape, and number adapted from [12].

The proposed effects of both polygenetic and environmental factors on hypodontia represented a paradigm shift in thinking with respect to the aetiology of tooth agenesis. Grahnén was first to count hypodontia as a hereditary anomaly and deemed that the transmission is determined by a dominant autosome, with incomplete penetrance and variable expressivity [46]. Later, Brook's theory claimed a significant association between tooth agenesis and microdontia, with sex differences in tooth size and number [27]. According to Brook, each anomaly occurred more frequently in first-degree relatives than in the population sample, and this suggested that the more severe the hypodontia was, the more likely the relatives were to also have hypodontia. Additionally, females were more likely to have hypodontia and microdontia, whereas males were more likely to have megadontia and supernumerary teeth and the model was later revised to clarify that both tooth size and shape are involved [12].  Figure 2 shows the aetiological model incorporating all of the multifactorial influences proposed.

Nowadays, most tooth agenesis theories recognise the complex nature of the genetic and environmental interactions involved in hypodontia. In fact, identification and gene sequencing in tooth morphogenesis are now possible due to genetic research advances, while understanding of the molecular mechanisms leading to tooth agenesis has also increased [5]. The following discussion will therefore focus on the specific genetic and environmental factors that have so far been linked to hypodontia.

### 4.3. Genetic Factors

Most craniofacial traits result from a complex interactions between genetic and environmental factors. Heritability can be expressed as a ratio that estimates the extent to which genetic characteristics affect the variation of a trait in a specific population at a point in time, and it is often investigated in twin studies [47]. It can range from 1 (complete genetic control) to zero (complete environmental control [47]) but can exceed theoretical thresholds if dominant gene effects and acquired environmental effects are included [48]. Many studies have demonstrated a strong genetic influence in hypodontia. Twin and family studies have determined that agenesis of lateral incisors and premolars is inherited via an autosomal dominant gene, with incomplete penetrance and variable expressivity [7, 8, 13, 32, 49–52]. There is no consensus, however, on whether hypodontia is a result of a polygenetic or single gene defect [53], although the former appears to be largely supported in the literature [13, 27].

Since tooth development is under some degree of genetic control, it follows that hypodontia is also under genetic influence. For this reason, recent efforts have focused on identifying the specific genes that are involved in regulating tooth development. Past research has mainly relied on family studies to identify these genetic variants. Studies of mutant mice and cultured tissue explants have examined the expression of numerous genes involved in tooth development and provided insight into inductive signalling and hierarchies of downstream transcription factors necessary for tooth development [54]. Over 300 genes are expressed and involved in tooth morphogenesis, including* MSX1, PAX9, AXIN2, EDA, SPRY2, TGFA, SPRY4, WNT10A, FGF3, FGF10, FGFR2,* and* BMP4* [23, 55, 56]. Among these genes,* PAX9 *(paired box gene 9),* MSX1 *(muscle segment homeobox 1),* AXIN2 *(axis inhibition protein 2), and* EDA *(ectodysplasin A) are the most frequently reported genes associated with nonsyndromic hypodontia [6, 57–60]. These all have roles in both signalling pathways and in mediating the signal transduction cascades [56].

*PAX9* is a transcription factor expressed in the tooth mesenchyme during tooth morphogenesis [60], with mutations in this gene being implicated in arresting tooth development at the bud stage. Heterozygous mutations in* PAX9, *in humans, have been associated with nonsyndromic tooth agenesis [2]. Most recently, a case-control study of 306 unrelated Portuguese individuals found that single nucleotide polymorphisms in the* PAX9* gene were associated with a high risk of maxillary lateral incisor agenesis [56].

*MSX1* is a member of the homeobox genes and it is expressed in regions of condensing ectomesenchyme in the tooth germ [61].* MSX1* gene mutations have been associated with premature termination of tooth development in animals [2, 21] and severe forms of hypodontia in humans. Recently, however, a frameshift mutation in* MSX1 *has been identified in a family missing all second premolars and mandibular central incisors [62].

The* AXIN2* gene is involved in cell growth, proliferation, and differentiation. It is a negative regulator of the* Wnt *signalling pathway, and this has been associated with lower incisor agenesis [23, 63]. In fact, these genes are involved in several forms of hypodontia, including syndromes in which this condition is a common feature [4].

More recently,* EDA* was found to be involved in isolated hypodontia. Mutations in this gene cause X-linked hypohidrotic ectodermal dysplasia (*HED*), which is characterised by sparse hair, fewer and smaller teeth, and a lack of sweat glands [42]. The* EDA* gene encodes a protein that is part of the tumour necrosis factor (*TNF*) family of ligands. Several studies have reported sporadic hypodontia in families affected by mutations in* EDA* and* EDA* receptor genes [64].* EDA* has also been shown to be involved in missing maxillary lateral incisor cases [56].

### 4.4. Environmental Factors

Craniofacial bones, cartilage, nerves, and connective tissue all originate from neural crest cells. Specific developmental cascades are therefore common to the morphogenesis of both teeth and some craniofacial structures [1]. Indeed, several syndromes involving hypodontia often exhibit various dysplasias and clefts. Environmental factors have long been known to be associated with a higher risk of some of these craniofacial anomalies. Factors such as trauma, infection, and toxins have been implicated [65].

Several studies have suggested that intrauterine conditions could be involved in the aetiology of hypodontia, such as with thalidomide. It was reported that hypodontia was more common in children with thalidomide embryopathy (7.7%) than in normal children (0.4%) [65, 66]. Chemotherapy and radiotherapy treatment in early infancy have also been implicated in the development of hypodontia [5, 67]. According to some research, rubella infection during pregnancy can cause hypodontia in the developing child [68]. Interestingly, however, maternal health during pregnancy was found to be unrelated to the expression of hypodontia [69]. Trauma, such as fracture of the alveolar process, may also contribute to hypodontia, though disruption of tooth germ development, although evidence supporting this is weak in the literature.

Neural crest cells are extremely sensitive to high levels of oxidative stress that can arise due to both genetic and environmental factors. It is generally accepted that oxidative stress in the form of smoking, for example [70], plays a central role in the development of neural crest cells and the aetiology of craniofacial anomalies. In fact, maternal smoking has been associated repeatedly with a higher risk of CLP [71]. Exposure to alcohol has also been suggested as a risk factor, and, although the evidence has been more inconsistent, some studies have reported that “binge” drinking patterns during pregnancy increase the risk for CLP [72]. Given that hypodontia shares similar molecular pathways with some craniofacial anomalies, it would be useful to investigate whether there is an association between environmental factors and hypodontia. Unfortunately, no study to date has investigated smoking and alcohol as risk factors for hypodontia. Indeed, the identification of environmental risks (particularly if they can be combined with genetic covariates) provides the best opportunity for prevention.

## 5. Psychosocial and Functional Impact

Oral-health-related quality of life (OHRQoL) measures are often used to assess the impact of malocclusion on health and well-being. They aim to assess the functional, psychological, and social implications of the condition on an affected individual. Although numerous studies in the literature report on the prevalence, aetiology, and treatment of hypodontia, only few have investigated OHRQoL in individuals with hypodontia [73]. The few studies that have been carried out provide some evidence that hypodontia may have an adverse impact on quality of life.

In a retrospective study of 451 patients with hypodontia, the most common patient complaints included spacing between the teeth, poor aesthetics, and awareness of missing teeth [19]. The authors suggested that delayed referral of the patient is likely to have a negative impact on the social and educational development of these patients. Locker and coworkers reported similar findings, although the affected children had oligodontia [74]. Interestingly, Laing and colleagues found that the extent of the patients' complaints was associated with the severity of the condition and the number of missing permanent teeth. Those who had no complaints at the time of presentation had retained primary teeth that masked the problem [75].

Functionally, individuals with hypodontia tend to have deeper bites and spaces. Missing posterior teeth may not only result in further deepening of the bite, but the condition may also lead to nonworking interferences, poor gingival contours, and overeruption of the opposing teeth. Moreover, patients with hypodontia have been found to experience more difficulty in chewing due to a smaller occlusal table. In a recent cross-sectional study, it was found that hypodontia patients have more chewing difficulties if the deciduous teeth associated with the missing permanent teeth had been exfoliated [75]. It is therefore plausible that hypodontia may pose functional limitations that affect an individual's general well-being and quality of life in the process, although there is currently limited evidence to support this.

Ultimately, hypodontia carries an aesthetic, functional, psychosocial, and financial burden for affected individuals [3]. For these patients, hypodontia is a lifetime problem, which requires careful treatment planning in order to ensure best treatment outcomes. Treatment plans also involve long-term maintenance [24] and family counselling. Meanwhile, treatment of hypodontia patients often takes a number of years, from their initial visit through to completion of treatment.

Most important is the assessment of the complaints of the patients and the parents. Treatment plans needed to manage the missing teeth of hypodontia patients are complex and require an interdisciplinary approach, which usually comes at a financial cost to both the patient and their family [24]. Because of this, an experienced team of dental specialists should be involved in the treatment process [5, 29].

## 6. Timely Management of Hypodontia

The restoration of spacing that results from the agenesis of missing teeth is frequently complicated by the remaining present teeth, which are in unfavourable positions. Nevertheless, orthodontic treatment can facilitate any restorative treatment that may be required. Common issues faced in treating hypodontia patients include space management, uprighting and aligning teeth, management of the deep overbite, and retention [33]. Space issues within the dental arch are multifactorial in origin. The amount of spacing is influenced by the presence of microdontia, retention of the primary teeth, and the abnormal eruptive paths and drifting of the successional teeth [24]. The decision on whether the treatment plan involves space closure or opening of the spaces of the missing mandibular second premolar depends on factors such as age of the patient; degree of inherent crowding; state of the deciduous teeth; type of malocclusion; and the circumstances of the patient (finances, attitude towards treatment, etc.).

In hypodontia patients, dental development is often delayed, as is orthodontic treatment [76, 77]. In young patients with mild crowding, extractions of specific primary teeth in the early mixed dentition may be useful to permit some favourable movement of adjacent teeth. However, evidence shows that space closure and alignment, in missing premolar cases for example, are often incomplete following such an interceptive measure, and further intervention may be necessary [24, 78]. This is supported by an earlier study, which reported that there was a residual space of 2 mm in the mandible after extraction of the primary second molars [79]. Conversely, it has been shown that extracting primary second molars at a suitable time, for example, before or close to the pubertal growth spurt peak, can lead to relief of anterior crowding and spontaneous closure of the missing permanent second premolar space [80]. It was concluded that space closure occurred by mesial/rotational movements and tipping of the first molars as well as distal movement of the first premolars [80]. It was also suggested that extractions did not impact the overjet, overbite, or incisor inclination [80]. The study lacked a sufficient sample size, with only 11 subjects studied; and inclusion criteria involved only subjects with normal occlusion.

The best time for orthodontic treatment of patients with agenesis of mandibular second premolars is usually early adolescence. This is when most of the remaining developing permanent teeth are erupting and most of the facial growth has happened [33]. Notably, more adults are seeking orthodontic treatment. The management of adults missing mandibular second premolars is often complicated by caries and periodontal disease as well as the lack of facial growth potential, which reduces their adaptation to occlusal disturbances [33].

## 7. Summary

Hypodontia is the most common craniofacial malformation in humans, as it may occur as part of a recognised genetic syndrome or as a nonsyndromic isolated trait. The most commonly missing teeth are the mandibular second premolars and the maxillary lateral incisors. While it is not known whether individuals with hypodontia have characteristic skeletal features and growth patterns, several clinical features are commonly seen, including microdontia, transposition of permanent teeth, ectopic permanent teeth, and infraocclusion of primary molar teeth [81]. Recent research suggests that both genetic regulation and environmental factors are involved in the aetiology of this condition, with the former playing a more important role [81]. Finally, it is also likely that specific hypodontia pathways have some effect on the function and psychosocial well-being of an individual, given the aesthetic, functional, and financial burden for affected individuals [81].
